# m^6^A-binding proteins: the emerging crucial performers in epigenetics

**DOI:** 10.1186/s13045-020-00872-8

**Published:** 2020-04-10

**Authors:** Yanchun Zhao, Yuanfei Shi, Huafei Shen, Wanzhuo Xie

**Affiliations:** Department of Hematology, the First Affiliated Hospital of Medical School of Zhejiang University, No. 79 Qingchun Road, Hangzhou, 310003 Zhejiang China

**Keywords:** N^6^-methyladenosine, m^6^A-binding proteins, Cancer, Virus, Immunity, Adipogenesis

## Abstract

N^6^-methyladenosine (m^6^A) is a well-known post-transcriptional modification that is the most common type of methylation in eukaryotic mRNAs. The regulation of m^6^A is dynamic and reversible, which is erected by m^6^A methyltransferases (“writers”) and removed by m^6^A demethylases (“erasers”). Notably, the effects on targeted mRNAs resulted by m^6^A predominantly depend on the functions of different m^6^A-binding proteins (“readers”) including YT521-B homology (YTH) domain family, heterogeneous nuclear ribonucleoproteins (HNRNPs), and insulin-like growth factor 2 mRNA-binding proteins (IGF2BPs). Indeed, m^6^A readers not only participate in multiple procedures of RNA metabolism, but also are involved in a variety of biological processes. In this review, we summarized the specific functions and underlying mechanisms of m^6^A-binding proteins in tumorigenesis, hematopoiesis, virus replication, immune response, and adipogenesis.

## Introduction

Epigenetic abnormalities, such as DNA methylation, histone modification, genomic imprinting, and chromosome remodeling, mainly affect the characteristics and functions of genes through regulating the transcription or translation processes [1], without altering the DNA sequences. These changes in gene expression are stable during cell self-renewal, division, and differentiation. Over the past decades, more and more attention has been paid to RNA modification with the help of high-throughput sequencing. To date, more than 100 types of modifications have been confirmed in various RNAs, including messenger RNAs (mRNAs), transfer RNAs (tRNAs), ribosomal RNAs (rRNAs), microRNAs (miRNAs), and long non-coding RNAs (lncRNAs). Notably, N^6^-methyladenosine (m^6^A), a well-known post-transcriptional modification first discovered and proposed in 1974, has been regarded as the most prevalent methylation in eukaryotic mRNAs [2, 3]. It is estimated that about 0.1–0.4% of all adenines are specially modified by m^6^A in mRNAs [4]. m^6^A usually appears at the RRACH sequences (R = A, G, or U; R = A or G; and H = A, C, or U) [5, 6], and mostly enriches in the 3′ untranslated regions (3’ UTRs) and near stop codons [7].

The regulation of m^6^A modification is dynamic and reversible (Table 1). It is established by m^6^A methyltransferases (also called “writers”), such as methyltransferase-like protein 3 (METTL3) [8, 9], METTL14 [8, 9] and Wilms tumor 1-associated protein (WTAP) [10]. And it is removed by m^6^A demethylases (also called “erasers”), containing fat-mass and obesity-associated protein (FTO) [16] and α-ketoglutarate-dependent dioxygenase alkB homolog 5 (ALKBH5) [17]. More importantly, the effects of m^6^A modification on RNA metabolism predominantly depend on the recognition by different m^6^A-binding proteins (also called “readers”), including but not limited to YT521-B homology (YTH) domain family, heterogeneous nuclear ribonucleoproteins (HNRNPs), and insulin-like growth factor 2 mRNA-binding proteins (IGF2BPs).

**Table 1:** The specific function of each m^6^A-related enzyme

Categories	m^6^A-related enzymes	Location	Mechanism	References
m^6^A writers	METTL3	Nucleus	Catalyzing methyl-group transfer	[8, 9]
	METTL14	Nucleus	Forming a heterodimer with METTL3 and strengthening its catalytic activity	[8, 9]
	WTAP	Nucleus	Promoting METTL3-METTL14 complex localization to nuclear speckles and modulating their recruitment to RNA targets	[10]
	VIRMA	Nucleus	Preferentially mediating m^6^A modification in the 3’UTR and near stop codon, and affecting the selection of methylation sites	[11]
	RBM15/RBM15B	Nucleus	Mediating m^6^A methylation of lncRNA XIST	[12]
	ZC3H13	Nucleus	Inducing the nuclear localization of Zc3h13-WTAP- Virilizer-Hakai complex	[13]
	METTL16	Nucleus	Functioning as a conserved U6 snRNA methyltransferase and regulating the abundance of intracellular SAM	[14, 15]
m^6^A erasers	FTO	Nucleus	Abrogating the m^6^A levels of targeted RNA via the oxidative demethylation activity	[16]
	ALKBH5	Nucleus	Removing the m^6^A modification of nuclear RNA	[17]
m^6^A readers	YTHDF1	Cytoplasm	Augmenting RNA translation through interacting with the translation initiation factor eIF3	[18]
	YTHDF2	Cytoplasm	Promoting RNA degradation by recruiting the CCR4-NOT deadenylase complex	[19, 20]
	YTHDF3	Cytoplasm	Not only promoting the translation of methylated RNA in cooperation with YTHDF1, but also strengthening RNA decay mediated by YTHDF2	[21, 22]
	YTHDC1	Nucleus	Mediating alternative splicing, facilitating m^6^A-methylated RNA nuclear export, and promoting X chromosome genes transcriptional silencing mediated by XIST	[12, 23, 24]
	YTHDC2	Cytoplasm	Increasing the translation efficiency of RNA	[25, 26]
	HNRNPA2B1	Nucleus	Accelerating the processing of primary miRNA, regulating alternative splicing, and acting as a “m^6^A-switch”	[27, 28]
	HNRNPC	Nucleus	Participating in the pre-mRNA processing and functioning as a “m^6^A-switch”	[29, 30]
	HNRNPG	Nucleus	Modulating pre-mRNA alternative splicing and acting as a “m^6^A-switch”	[31]
	IGF2BP1	Cytoplasm	Fortifying RNA stability	[32]
	IGF2BP2	Cytoplasm	Increasing the stability of RNA	[32]
	IGF2BP3	Cytoplasm	Facilitating RNA stabilization	[32]

The typical m^6^A methyltransferase complex mainly consists of METTL3, METTL14, and WTAP. METTL3, a core component with methyltransferase activity, can combine with S-adenosyl methionine (SAM) and then mediate RNA methylation in the nucleus [8, 9]. However, METTL14 is not a subunit to directly catalyze the methyl-group transfer. It functions as an RNA-binding platform to form a stable heterodimer with METTL3, eventually strengthening the catalytic effect of METTL3 [8, 9]. Interestingly, WTAP interacts with METTL3-METTL14 complex to not only promote their localization to nuclear speckles, but also modulate their recruitment to mRNA targets [10]. Moreover, there are a lot of regulatory factors binding to the catalytic complex, such as vir-like m^6^A methyltransferase associated (VIRMA, also termed as KIAA1429), RNA-binding motif protein 15/15B (RBM15/RBM15B), and zinc finger CCCH domain-containing protein 13 (ZC3H13). VIRMA preferentially mediates m^6^A modification in the 3′ UTR and near stop codon, affecting the selection of methylation sites [11]. RBM15/RBM15B play an important role in the X-inactivation and gene silencing through mediating the m^6^A methylation of lncRNA XIST [12]. ZC3H13 induces the nuclear localization of Zc3h13-WTAP-Virilizer- Hakai complex to regulate m^6^A methylation and trigger the self-renewal of embryonic stem cells [13]. It is noteworthy that novel methyltransferases are gradually discovered. For instance, METTL16, a conserved U6 snRNA methyltransferase, participates in the regulation of intracellular SAM abundance via methylating the SAM synthetase gene MAT2A and controlling its intron retention [14, 15].

Currently, only two demethylases have been identified, which both belong to AlkB family proteins. The first one is FTO that is located in the nucleus and abrogates the m^6^A levels via the oxidative demethylation activity [16]. Moreover, another demethylase ALKBH5 can also remove the m^6^A modification of nuclear RNA, and further modulate nuclear RNA export, RNA metabolism, and gene expression [17].

The YTH domain is found in 174 different proteins in eukaryotes [33], with a size from 100 to 150 amino acids. It is featured by 14 invariant and 19 highly conserved residues [34], and contains a structure of four α helices and six β strands [35]. Interestingly, the six β strands shape a β barrel and then stabilize the hydrophobic core through combining with α helices [35]. The recognized YTH domain family consists of YTH domain family protein 1-3 (YTHDF1-3, DF family) and YTH domain containing protein 1-2 (YTHDC1-2, DC family). YTHDF2, the first identified m^6^A reader, promotes mRNAs degradation and reduces the stability of targeted transcripts through recruiting the CCR4-NOT deadenylase complex [19, 20]. On the contrary, YTHDF1 augments mRNAs translation by interacting with the translation initiation factor eIF3 rather than by the m^7^G-cap-dependent manner [18]. Intriguingly, it has been reported that there are a great number of common targets that YTHDF3 shares with YTHDF1 and YTHDF2 [21]. Functionally, YTHDF3 not only cooperates with YTHDF1 to promote the translation of methylated mRNAs, but also strengthens mRNAs decay mediated by YTHDF2, showing a cooperative relationship among YTHDF proteins. YTHDF3 recognizes the m^6^A-containg mRNAs and increases their expression through combining with 40S and 60S ribosomal subunits [21, 22]. Moreover, YTHDF3 can remarkably facilitate protein synthesis of YTHDF1/3 common targets, but not YTHDF3 unique targets. Notably, YTHDF3 depletion decreases the binding of YTHDF1 and YTHDF2 to their target transcripts, while YTHDF1 or YTHDF2 loss reduces the amount of RNA that is bound by YTHDF3 [21]. It shows the important role of YTHDF3 in the RNA binding specificity and YTHDF1/2 in the RNA binding affinity. YTHDC1 is widely distributed in the nucleus and its YTH domain is in complex with the RNA 5-mer GG(m^6^A)CU to form a crystal structure [35]. The 3′ terminal nucleotides are stabilized via the interactions of cation-π-π; however, the 5′ terminal nucleotides remain flexible [36]. The function of YTHDC1 is to mediate alternative splicing by recruiting RNA splicing factor SRSF3 and blocking SRSF10 from binding to mRNAs [23]. YTHDC1 also interacts with SRSF3 and NXF1 to facilitate the m^6^A-methylated mRNAs nuclear export [24]. Furthermore, YTHDC1 plays a promoting role in the X chromosome genes transcriptional silencing mediated by XIST [12]. YTHDC2, a putative RNA helicase, contains YTH domain, helicase domain, R3H domain, and ankyrin repeats [37], which is of great significance to increase the translation efficiency of mRNAs [25, 26].

Besides, there are members of HNRNP family who have the potential to identify the m^6^A modification of mRNAs, including heterogeneous nuclear ribonucleoproteins A2/B1 (HNRNPA2B1), heterogeneous nuclear ribonucleoproteins C (HNRNPC) and heterogeneous nuclear ribonucleoproteins G (HNRNPG). The binding of HNRNP proteins to m^6^A sites can be enhanced via the mRNAs structural alteration induced by m^6^A methylation [31, 38, 39], which is called as “m^6^A-switch”. HNRNPA2B1 is a nuclear m^6^A-binding protein that accelerates the processing of primary miRNAs (pri-miRNAs) through interacting with DGCR8 protein in an m^6^A-dependent manner [27, 28]. Moreover, HNRNPA2B1 possesses a capability of modulating the alternative splicing of transcripts [27]. HNRNPC, a RNA-binding protein in nucleus, plays an important role in the pre-mRNAs processing [29, 30]. HNRNPG is an m^6^A reader that selectively binds RNAs via Arg-Gly-Gly (RGG) motifs in the low-complexity region [31]. HNRNPG modulates pre-mRNAs alternative splicing by interacting with both the phosphorylated carboxy-terminal domain of RNA polymerase II (RNAPII) and m^6^A-methylated pre-mRNAs in a RGG-region-dependent manner [31].

Additionally, IGF2BPs are the distinct and conserved m^6^A-binding proteins, whose RNA-binding domains consist of four K homology (KH) domains and two RNA recognition motif (RRM) domains [40]. However, only the third and fourth KH domains (KH3-4) are indispensable in recognizing the m^6^A sites of mRNAs. Functionally, IGF2BP1-3 fortify the stability and increase the translation efficiency of m^6^A-modified mRNAs [32]. And the mRNA stabilization mediated by IGF2BPs may be strengthened via recruiting the co-factors of IGF2BP1-3, including ELAV-like RNA binding protein 1 (ELAVL1, also called HuR) and matrin 3 (MATR3) [32].

In a word, m^6^A methylation participates in multiple procedures throughout the life cycle of mRNAs, such as alternative splicing, translation, translocation, and degradation. Recent years have witnessed a remarkable advance in understanding the roles of m^6^A modification in a variety of biological processes, particularly m^6^A readers. Herein, we provided an updated review to summarize the functions and mechanisms of m^6^A-binding proteins in tumorigenesis, hematopoiesis, viral replication, immunity, and adipogenesis.

### The role of m^6^A-binding proteins in human solid cancers

#### Hepatocellular carcinoma (HCC)

Hypoxia is a common characteristic in various solid cancers, acting as a promoter in tumorigenesis via the regulation by hypoxia-inducible factor (HIF) [41]. A study reported by Zhong and colleagues showed that HIF1α-dependent hypoxia downregulated the expression of YTHDF2 in HCC cells, and that YTHDF2 depletion promoted tumor cells proliferation [42]. Mechanistically, EGFR, the upstream transcript of ERK/MAPK pathway, was modified by m^6^A and recognized by YTHDF2. Then YTHDF2 destabilized EGFR by facilitating mRNA decay, which in turn suppressed the ERK/MAPK signaling pathway. However, Hou et al. found that HIF2α-mediated hypoxia abrogated YTHDF2 expression in HCC cells, and that YTHDF2 deficiency facilitated inflammation, vasculature reconstruction, and metastasis through decreasing the degradation of m^6^A-marked interleukin-11 (IL-11) and serpin family E member 2 (SERPINE2) mRNAs which were two crucial factors in the processes of inflammation-induced malignancy and vascular remodeling [43]. SOCS2, a negative regulator of JAK/STAT pathway, was identified as a tumor suppressor of HCC. There was a direct binding between YTHDF2 and the m^6^A sites of SOCS2 mRNA in HCC [44]. Knockdown of YTHDF2 remarkably decreased the silencing of SOCS2 and increased its expression.

Notably, the function of YTHDF1 in the progression of HCC has also been reported. Epithelial-mesenchymal transition (EMT), a critical step of tumor cells metastasis, is a process in which epithelial tumor cells lose junction proteins and cell polarity [45, 46]. And Snail is a key transcription factor of EMT. In HCC cells, YTHDF1 could dramatically accelerate the translation of Snail mRNA via an m^6^A-dependent manner, thus contributing to tumor metastasis [47].

Additionally, Huang et al. demonstrated that IGF2BPs could preferentially recognize and bind to the m^6^A sites at the coding region instability determinant (CRD) of oncogene MYC in HCC [32]. Loss of IGF2BPs substantially reduced the expression of MYC mRNA and then inhibited HCC cells proliferation, migration, and colony formation ability. Furthermore, serum response factor (SRF) could promote cell proliferation, invasion, and metastasis of HCC. And IGF2BP1 increased SRF expression in a conservative manner, which was strictly dependent on the m^6^A modification at the 3′ UTR [48].

#### Colorectal cancer (CRC)

There was a study exploring the relationship between YTHDF1 and CRC. In CRC, YTHDF1 could promote cell proliferation and invasion [49]. Silencing of YTHDF1 significantly downregulated the expression of cancer stem cell markers but notably upregulated the enterocyte markers expression, suggesting that YTHDF1 was involved in modulating the stem cell-like activity in CRC. Mechanistically, YTHDF1 took advantage of the m^6^A-dependent mRNA translation to not only increase the β-catenin expression and its nuclear signaling activity, but also facilitate the expression of downstream targets of Wnt/β-catenin pathway, including WNT6 and FZD9 [49].

Interestingly, Ni et al. discovered a negative functional loop of lncRNA GAS5/YAP/YTHDF3 axis in CRC [50]. GAS5 could promote YAP to translocate from nucleus to cytoplasm, and facilitate YAP phosphorylation, ubiquitination and degradation, thus inhibiting CRC progression. And YAP targeted at the promoter of YTHDF3, leading to an increase of YTHDF3 expression. Then, YTHDF3 recognized m^6^A-containing GAS5 and accelerated its degradation, further affecting the activity of YAP signaling pathway in CRC [50].

Moreover, circNSUN2 is a circular RNA that facilitates CRC cells metastasis and aggressiveness, and contains m^6^A methylation at the exon 5-exon 4 junction site [51]. On the one hand, YTHDC1 promoted circNSUN2 export from nucleus to cytoplasm. On the other hand, the KH3-4 di-domain of IGF2BP2 interacted with circNSUN2 and then form a circNSUN2/IGF2BP2 complex to fortify the stability of HMGA2 that was a RNA inducing EMT and promoting CRC liver metastasis [51]. In addition, SOX2, an important gene to promote tumor initiation and metastasis, exhibited an elevated m^6^A level [52]. And IGF2BP2 could directly bind to the methylated SOX2 and dramatically enhance the stability of SOX2 mRNA by preventing it being degraded.

#### Gastric cancer (GC)

In GC, SEC62 overexpression promoted cell proliferation and inhibited cell apoptosis. And IGF2BP1 specially recognized the m^6^A-modified SEC62 mRNA and augmented its expression at both mRNA and protein levels [53]. Furthermore, another member of IGF2BP proteins IGF2BP3 could directly bind to the m^6^A sites of HDGF mRNA and then improved HDGF stability by enhancing the m^6^A-associated mRNA translation [54]. And HDGF has an ability of facilitating tumor angiogenesis. Knockdown of IGF2BP3 markedly reduced the expression of HDGF mRNA and suppressed tumor growth in GC.

#### Lung cancer

Pentose phosphate pathway (PPP) that is catalyzed by glucose-6-phosphate dehydrogenase (G6PD) and 6-phosphogluconate dehydrogenase (6PGD) provides tumor cells the ribose-5-phosphate and NADPH. In lung cancer, YTHDF2 depletion could increase lactate production, and decrease oxidative PPP flux and NADPH/NADP^+^ ratio [55], indicating that YTHDF2 participated in lung cancer cells metabolism and affected tumor cells growth. Mechanistically, YTHDF2 significantly increased the translation of 6PGD, which was dependent on the m^6^A methylation sites of 6PGD mRNA.

Intriguingly, a recent research reported the different expression levels of YTHDF1 during disparate circumstances [56]. YTHDF1 was upregulated under normoxia but downregulated under hypoxia conditions. When non-small cell lung cancer (NSCLC) cells were in a normoxia state, overexpression of YTHDF1 promoted tumor cells proliferation and xenograft tumor formation by augmenting the translations of m^6^A-marked CDK2, CDK4, and cyclinD1, three key regulators in the G0/G1 cell cycle transition. However, when NSCLC cells were treated with cisplatin, a chemotherapy drug inducing the reactive oxygen species (ROS) accumulation, YTHDF1 was decreased and led to cisplatin resistance in tumor cells via regulating the Keap1-Nrf2-AKR1C1 axis [56].

Notably, m^6^A-binding proteins are observed to be associated with hippo signaling pathway effector YAP/TAZ. Jin et al. demonstrated that YTHDF1/3 complex, together with eIF3b, recognized m^6^A-containing YAP and upregulated its expression level through interacting with the translation initiation complex, thus boosting NSCLC cells growth, invasion, and migration [57]. Moreover, MALAT1 which was modified by m^6^A and then stabilized by the METTL3/YTHDF3 complex, sponged miR-1914-3p to further promote YAP expression [57]. Another important finding was that METTL3 facilitated lung cancer cells growth and invasion by functioning like an m^6^A reader [58]. METTL3 accelerated the translation of m^6^A-marked EGFR and TAZ through directly recruiting eIF3 to the translation initiation complex rather than relying on the methyltransferase activity or other m^6^A readers.

#### Bladder cancer (BC)

ITGA6 acted as a tumor promoter to facilitate BC cells growth and progression in vitro and in vivo. m^6^A enriched at ITGA6 mRNA, and was preferentially bound by YTHDF1 and YTHDF3 [59]. Knockdown of YTHDF1 and YTHDF3 abrogated the expression of ITGA6. Another study showed that BC-related oncogene CPCP1 could be marked by m^6^A and selectively recognized by YTHDF1 [60]. And YTHDF1 remarkably increased CDCP1 expression. Interestingly, in addition to catalyzing CDCP1 methylation, METTL3 also could bind to the m^6^A sites of CDCP1 and facilitate its translation, playing a role like an m^6^A-binding protein. Furthermore, METTL3 could accelerate the binding of YTHDF1 to CDCP1. Loss of METTL3 and YTHDF1 produced a cooperative effect on decreasing the expression of CDCP1 [60].

#### Endometrial cancer

In endometrial cancer, YTHDF1 and YTHDF2 regulated the expression of critical enzymes in the AKT signaling pathway via different ways [61]. PHLPP2 is a negative regulator of AKT pathway. YTHDF1 promoted the m^6^A-mediated translation of PHLPP2 through strengthening its binding to actively transcribing ribosomes. However, YTHDF2-mediated decay diminished the abundance of PRR5, PRR5L, and mTOR, which were three key components of mTORC2 complex, a positive regulator of AKT pathway.

#### Ovarian cancer

EIF3C is a subunit of EIF3 which is indispensable for protein synthesis. YTHDF1 was highly expressed in ovarian cancer and targeted at EIF3C to enhance its translation efficiency via an m^6^A-dependent manner; therefore, affecting the overall translational output and regulating tumor cells proliferation, migration, and invasion [62].

#### Cervical cancer (CC)

LncRNA GAS5-AS1 diminished the m^6^A level of GAS5 by interacting with m^6^A demethyltransferase in cervical cancer, and then decreased GAS5 degradation and increased GAS5 stability via reducing the YTHDF2-mediated decay, eventually resulting in the suppression of cancer tumorigenicity and metastasis [63].

#### Melanoma

In melanoma, depletion of YTHDF2 accelerated cell proliferation and migration by strikingly upregulating the mRNA levels of three key intrinsic pro-tumorigenic factors, including PD-1 (PDCD1), CXCR4, and SOX10, which was dependent on a reduced m^6^A-mediated mRNA decay [64]. Furthermore, YTHDF1 promoted the translation of tumor suppressor HINT2 mRNA which was methylated by m^6^A, thus playing a repressive role in ocular melanoma [65].

#### Breast cancer

BNIP3 is a pro-apoptosis gene. And YTHDF2 could recognize the m^6^A sites of BNIP3. As a result, the degradation of BNIP3 mRNA was increased and its expression was decreased, promoting the growth of tumor cells [66].

#### Pancreatic cancer

DANCR can facilitate cell proliferation and stemness-like properties. And IGF2BP2 targeted at m^6^A-containing DANCR to enhance its translation. Then, IGF2BP2 together with DANCR jointly boosted the tumorigenesis of pancreatic cancer [67].

### The role of m^6^A-binding proteins in hematopoiesis

Hematopoiesis is an intricately detailed process in which immature hematopoietic stem cells (HSCs) differentiate into cellular components of peripheral blood, including leukocytes, erythrocytes, and platelets [68]. HSCs possess not only a capability of self-renewal but also a potential of multilineage differentiation, thus maintaining the homeostasis of hematological system. The sources of HSCs are commonly bone marrow (BM), umbilical cord blood (UCB), and mobilized peripheral blood (MPB) [69]. Moreover, HSCs are characterized by a variety of surface markers, such as CD34^+^, CD90^+^, Lin^−^, CD38^−^, and CD45RA^−^ [70, 71].

Hematopoietic stem and progenitor cells (HSPCs) are initially derived from endothelial cells via a process called endothelial-to-hematopoietic transition (EHT) [72]. In EHT, Notch1 signaling pathway represses HSPCs programming and further influences the generation of HSPCs [73, 74]. A Chinese study first demonstrated that YTHDF2 regulated the production of earliest HSPCs during vertebrate embryogenesis [75]. YTHDF2 recognized the m^6^A peaks near the stop codons of arterial endothelial gene notch1a and promoted its mRNA decay, which in turn contributed to the inhibition of Notch1 signaling and modulated the development of HSPCs.

Besides, m^6^A-binding proteins are of great importance to HSCs expansion. Li et al. found that YTHDF2 knockout led to a dramatic increase in the numbers of both long-term HSCs and short-term HSCs, but not the progenitors and lineage cells [76]. Mechanistically, YTHDF2 facilitated the decay of m^6^A-modified mRNAs, such as TAL1, RUNX1, STAT5, and GATA2, which encoded transcription factors related with the self-renewal of stem cells. Furthermore, knockdown of YTHDF2 significantly promoted the stem cells maintenance and the human umbilical cord blood (hUCB) HSCs expansion in vitro [76]. Similarly, Wang et al. confirmed that the mRNAs clearance induced by YTHDF2 via an m^6^A-dependent manner was involved in the regeneration of HSCs [77]. Under transplantation and hematopoietic stress conditions, depletion of YTHDF2 increased the number of HSCs by reducing the degradation of Wnt signaling pathway downstream targets.

There is no doubt that the disorder of hematopoiesis is strongly associated with a lot of human diseases, including lymphoid and myeloid hematologic malignancies. Malignant hematopoiesis not only breaks the balance between self-renewal and differentiation of HSCs, but also puts the progenitor cells at a risk of developing leukemia [78]. So far, studies about the role of m^6^A readers in hematological neoplasms mainly focus on acute myeloid leukemia (AML). AML is regarded as a clonal hematopoietic dysregulation in which leukemic stem cells (LSCs) retain the self-renewal capacity, but lose the myeloid differentiation capacity [79, 80]. Paris and colleagues observed that YTHDF2 was overexpressed in AML and closely correlated with LSCs activity [81]. Functionally, YTHDF2 selectively compromised the initiation and propagation of AML, but does not impede normal hematopoiesis. Mechanistically, as a gene inhibiting the accumulation of leukemic cells, TNFR2 was marked by m^6^A. And its expression was regulated by the YTHDF2-mediated mRNA decay. In addition, silencing of YTHDF2 increased AML cells sensitivity to TNF-induced apoptosis. Su et al. revealed that YTHDF2 could recognize the m^6^A sites of MYC and CEBPA mRNAs in AML [82]. YTHDF2 loss facilitated the expression of MYC and CEBPA by increasing their stability, and also affected the sensitivity of leukemia cells to the tumor suppressor R-2-hydroxyglutarate (R-2HG).

### The role of m^6^A-binding proteins in viruses

#### Kaposi’s sarcoma-associated herpesvirus (KSHV)

KSHV has two phases called latent phase and lytic replication phase. Under immunosuppressive conditions, KSHV in the latent phase is reactivated to undergo lytic replication, then producing new virus. Most transcripts encoded by KSHV were modified by m^6^A. Hesser et al. found that m^6^A modification regulated the lytic viral gene expression in a cell type-specific manner [83]. In KSHV-infected renal carcinoma cell lines, loss of YTHDF2 blocked virus lytic cycle progression and virion production through reducing the expression of m^6^A-modified ORF50. However, in the KSHV-positive B cell line, ORF50 expression was increased when YTHDF2 was depleted. Therefore, the same YTHDF2-mediated m^6^A machinery may lead to either pro- or anti-viral effects depending on the cell context, while the underlying mechanism remains unknown and demands further study.

In addition, the m^6^A peaks of replication transcription activator (RTA), a critical protein associating with KSHV lytic switch, could be recognized by YTHDC1 with its related splicing factors SRSF3 and SRSF10, therefore enhancing the pre-mRNA splicing of RTA [84]. Interestingly, RTA itself could conversely increase the m^6^A levels and promote its own pre-mRNA splicing in an m^6^A-dependent manner, which further facilitated lytic gene expression and replication. Tan et al. revealed that YTHDF2 could bind to RTA, ORF57, and ORF-K8 lytic transcripts in KSHV, and decrease their expression and half-life via the m^6^A-associated RNA decay [85].

#### Human immunodeficiency virus (HIV)

Tirumuru et al. have established a genome-wide mapping of m^6^A modification within HIV-1 RNA in several HIV-1-infected cells. The m^6^A peaks predominantly located at the 3′ UTR, 5′ UTR and some coding genes of the HIV-1 genome [86]. During the reverse transcription phase, YTHDF1-3 proteins could bind to the m^6^A-modified HIV-1 RNAs and facilitate their degradation. Therefore, YTHDFs suppressed HIV-1 infection through blocking the viral reverse transcription. Moreover, m^6^A methylation of HIV-1 RNA modulated by m^6^A writers or erasers was crucial for the effective HIV-1 protein synthesis [86]. Then, the underlying mechanisms of YTHDFs-induced HIV-1 infection inhibition were further explored [87]. In infected target cells, it was identified that YTHDFs preferentially interacted with m^6^A-marked viral genomic RNA (gRNA) at 5′ UTR and abolished the level of gRNA. As a consequence, both early and late reverse transcription products were decreased, which contributed to the impairment of HIV-1 replication and infectivity. Meanwhile, in virus-producing cells, endogenous YTHDFs and HIV-1 Gag protein constituted a complex with RNAs, and expression of Gag was strengthened by YTHDFs. It was noteworthy that the suppression of HIV-1 reverse transcription and promotion of Gag processing were both triggered by YTHDFs, which implied the complicated roles of m^6^A in viral infection [87].

Additionally, there was another study also revealing the m^6^A editing profiling of HIV-1 genome [88]. They found that m^6^A sites were mainly enriched in 3′ UTR and that m^6^A residues were sufficient to enhance mRNA expression via recruiting YTHDFs. Surprisingly, YTHDFs positively regulated the HIV-1 protein expression and virus replication, which was contradictory to previous conclusions. This may be caused by discrepant cells used or various time course of virus infection. Anyhow, the functional complexity of YTHDFs on HIV-1 production and infectivity requires further investigations.

#### Influenza A virus (IAV)

m^6^A modification enriched at the IAV mRNAs encoding major structural proteins, including HA, NA, M1/M2, and NP, but not at the virus mRNAs encoding viral polymerase proteins, such as PB2, PB1, and PA [89]. And the binding targets of YTHDF2 in IAV were mainly at the transcripts encoding viral structural proteins. Overexpression of YTHDF2 significantly increased the expression of IAV NS1, M2, and NP proteins, and facilitated IAV replication, infectious particle production and viral spread [89].

#### Hepatitis C virus (HCV)

In HCV infection, YTHDFs relocalized to lipid droplets where viruses assembled, and specially bound to m^6^A residues within the E1 region which were functionally relevant [90]. YTHDFs negatively modulated HCV particle production, showing a significant role of m^6^A in affecting the life cycle of HCV.

### The role of m^6^A-binding proteins in immunity

Immune system has an ability to recognize and eliminate foreign antigenic substances via innate immune response and acquired immune response. Dendritic cells (DCs) are a class of professional antigen presenting cells (APCs) and play a key role in activating adaptive immune response and maintaining immune homeostasis. CCR7-induced DC migration was regulated by an lncRNA-associated feedback. To be specific, YTHDF2 could recognize the m^6^A-modified lnc-Dpf3 and promote its degradation. At the late stage after CCR7 stimulation, the m^6^A level of lnc-Dpf3 was diminished, which lead to a decreased YTHDF2-mediated mRNA decay of this lncRNA. Subsequently, enhanced lnc-Dpf3 in turn repressed DC migration by inhibiting HIF1α-dependent glycolysis, resulting in the suppression of immune response [91]. Moreover, it is well-characterized that CD40 and CD80 enhance DC antigen presentation and T cell activation, while Tirap induces TLR4/NF-κB signaling pathway. And they were all marked by METTL3-guided m^6^A modification. Then YTHDF1 interacted with CD40, CD80 and Tirap to facilitate their translation, thus contributing to the activation and maturation of DCs [92].

Recently, an emerging view of circRNAs-based innate immune system is proposed. Foreign circRNAs may function as powerful adjuvants to invoke antigen-specific T and B cell responses through activating RNA pattern recognition receptor RIG-I in the presence of K63-polyubiquitin (K63-Ubn). Nevertheless, endogenous human circRNAs would be modified by m^6^A methylation and bound by YTHDF2, which was capable of abrogating the activation of the RIG-I/K63-Ubn/circRNA complex. That is to say, YTHDF2 is crucial for the m^6^A-containing suppression of innate immunity [93].

#### Inflammatory response

Lipopolysaccharide (LPS) is the main component of the cell walls of gram-negative bacteria and can stimulate macrophages to secrete a number of inflammatory cytokines, such as IL-6, IL-1β, and TNF-α, through enhancing the activity of MAPK and NF-κB pathways [94]. YTHDF2 was upregulated in macrophages stimulated by LPS [95]. YTHDF2 silencing promoted the phosphorylation of p38 and ERK1/2 of MAPK signaling pathway and p65 of NF-κB signaling pathway by increasing MAP2K4 and MAP4K4 expression, which initiated the LPS-induced inflammatory response.

#### Antiviral immune response

Viruses have a feature of propagation, but it can be blocked by the type I interferon response and the expression of interferon-stimulated genes (ISGs) [96]. IFNB mRNA, which encoded interferon-β (IFN-β) to trigger type I interferon response, was methylated by m^6^A and bound by YTHDF2 [97]. YTHDF2 deficiency promoted the stability of IFNB and a constant IFN-β production, thereby leading to a strongly antiviral innate immune response and further decreasing the viral propagation. Similarly, a study conducted by Rubio et al. also showed that YTHDF1 and YTHDF2 play an antiviral role by modulating IFN in an m^6^A-recognition way [98]. During the DNA viruses infection, nucleus-localized HNRNPA2B1 recognized viral DNA and became dimerized. Then, it was demethylated at arginine-226 mediated by the arginine demethylase JMJD6 [99]. As a result, HNRNPA2B1 translocated from nucleus to cytoplasm and initiated the TBK1-IRF3 pathway, resulting in an increase of IFN-α/β production and the activation of type I interferon response. Interestingly, the constitutive association of hnRNPA2B1 and FTO was impaired after HSV-1 infection. Therefore, hnRNPA2B1 indirectly increased the m^6^A modification of bound transcripts including IFI16, cGAMP, cGAS, and STING. By regulating their nucleocytoplasmic trafficking and translation, hnRNPA2B1 further induced the activation of TBK1-IRF3 pathway.

#### Antitumor immune response

A few of antigens are considered to be expressed by tumor cells, mainly containing tumor-associated antigens (TAAs) and tumor-specific antigens (TSAs). Immune system can identify these foreign antigens to distinguish cancer cells from non-cancer cells and initiate antitumor immune responses. Recently, with the development of bioinformatic methods, the mysterious veil of tumor mutation-derived antigens (neoantigens) has been gradually uncovered. Neoantigens can induce antitumor immune responses and predict the responses to immunotherapy [100, 101]. Nevertheless, neoantigens stimulation does not produce a lasting T cell immune response to clear the tumor cells, thus resulting in the tumor immune escape.

m^6^A modification can modulate immune response induced by tumor neoantigens via YTHDF1. Functionally, absence of YTHDF1 specifically enhanced the cross-prime ability of DCs and further elicited CD8+ T cells responses [102]. Mechanistically, YTHDF1 recognized and bound to the m^6^A peaks of the transcripts encoding lysosomal proteases, markedly increasing the translation efficiency of lysosomal cathepsins in DCs but inhibiting the capability of cross-presentation of DCs. Notably, there were substantially increased neoantigen-specific CD8+ T cells when YTHDF1 was deficient. In addition, knockdown of YTHDF1 upregulated the expression of PD-L1 and improved the PD-L1 checkpoint blockade’s antitumor effect and the patients’ clinical outcomes [102].

### The role of m^6^A-binding proteins in adipogenesis

Obesity is an important public health problem, which can increase the risk of cardiovascular diseases, diabetes mellitus, and cancers [103]. Adipogenesis is a sophisticated process of cell differentiation in which pre-adipocytes become adipocytes. It is closely associated with obesity and is regulated via transcriptional cascade and extracellular signals [104]. Notably, increasing evidence reveals that m^6^A-binding proteins play a critical role in adipogenesis.

Firstly, m^6^A reader YTHDF2 can influence the process of mitotic clonal expansion (MCE). CCNA2 and CDK2 are two pivot cell cycle regulators, promoting cells from S phase to G2 phase. During adipogenesis, the m^6^A levels of CCNA2 and CDK2 decreased, but their expression increased [105]. Mechanistically, YTHDF2 recognized the methylated sites of CCNA2 and CDK2 and subsequently decayed them. Interestingly, epigallocatechin gallate (EGCG), a catechin in green tea, plays an anti-adipogenesis role. EGCG could increase not only the m^6^A modification of CCNA2 and CDK2 but also the expression of YTHDF2 [106]. And YTHDF2-mediated decay reduced the expression of CCNA2 and CDK2 mRNAs, thereby impairing adipogenesis. Moreover, another key cell cycle regulator CCND1 is also involved in the adipocytes differentiation. YTHDF2 preferentially bound to m^6^A-methylated CCND1 mRNA and diminished its expression, partially arresting MCE and inhibiting adipogenesis [107].

Secondly, m^6^A reader YTHDF2 can modulate JAK/STAT signaling pathway. Wu et al. observed that there was a negative relationship between the expression of JAK2 mRNA and m^6^A level in preadipocytes [108]. Further study found that JAK2 was a direct target of YTHDF2, and that knockdown of YTHDF2 remarkably increased JAK2 mRNA and protein levels via a decreased m^6^A-based mRNA decay, which led to an inactivation of JAK2/STAT3/C/EBPβ pathway and thus hindered adipogenesis. Similarly, in the porcine bone marrow stem cells (pBMSCs), m^6^A methylation was enriched at JAK1 mRNA, and JAK1 knockdown could suppress adipocytes differentiation [109]. YTHDF2 recognized JAK1 and then diminish its expression through enhancing mRNA degradation, consequently inhibiting the phosphorylation of STAT5 and the initiation of JAK1/STAT5/C/EBPβ pathway, and preventing BMSCs from differentiating into adipocytes.

Thirdly, m^6^A reader YTHDF2 can regulate the expression of adipogenic-related genes. Bmal1 is an important part of mammalian circadian clock gene regulatory networks, mainly regulating metabolism. PPARα is a critical transcription factor that modulates the expression of genes involved in lipid metabolism. Loss of Bmal1 markedly increased the m^6^A levels of PPARα mRNA [110]. And YTHDF2 could mediate the mRNA decay of PPARα to decrease its stability and lead to a reduction of lipid accumulation, suggesting a close relationship between circadian clock regulation, m^6^A modification, and lipid metabolism. Furthermore, FAM134B is a positive regulator of lipid deposition in preadipocytes, whose overexpression causes the enhancement in the expression of adipogenic differentiating factors PPARγ, CEBPα, and FAS. And FAM134B was featured by m^6^A modification. Then, YTHDF2 interacted with FAM134B and drive its mRNA degradation via an m^6^A-based mechanism, leading to an inhibition of adipogenesis [111]. Song et al. found that Zfp217 could accelerate the expression of key adipogenic genes PPARγ, aP2, LPL, and Adiponectin [112]. Interestingly, Zfp217 could activate the transcription of FTO, while YTHDF2 was identified to inhibit the demethylase activity of FTO. And Zfp217 interacted with YTHDF2 to block its suppression of the m^6^A demethylase, finally induces the abolished m^6^A level of adipogenic genes. Thus, YTHDF2-mediated decay of these genes was decreased, which facilitated adipogenic differentiation [112].

Fourthly, m^6^A reader YTHDF2 can interact with autophagy-related genes. YTHDF2 targeted at m^6^A-modified Atg5 and Atg7, and modulated their expression via the mRNA decay [113]. Depletion of YTHDF2 accelerated autophagy and adipogenesis through enhancing the stability of Atg5 and Atg7, suggesting that m^6^A modification might regulate adipocytes differentiation by autophagy.

In addition to YTHDF2, m^6^A-binding protein YTHDF1 also participates in the process of adipogenesis. Jiang et al. determined the whole transcriptome-wide m^6^A profiles of the longissimus dorsi muscles (LDMs) in lean-type and obese-type pigs and found a unique m^6^A-methylated gene in the obese-type pigs called MTCH2 that was involved in lipid accumulation and oxidation [114]. And YTHDF1 targeted at the m^6^A motif of MTCH2 mRNA to improve its translation efficiency and further promote adipogenesis. Another study revealed that UCP2 and PNPLA2 were two distinguished m^6^A-containing genes in fat pig model [115]. After the depletion of m^6^A modification, mutated UCP2 suppressed adipogenesis while mutated PNPLA2 facilitated lipid accumulation. Mechanistically, m^6^A methylation promoted PNPLA2 expression by YTHDF1-mediated translation, whereas inhibited UCP2 expression seemingly dependent on neither YTHDF1 nor YTHDF2 [115].

## Conclusions and perspective

**Fig. 1:**
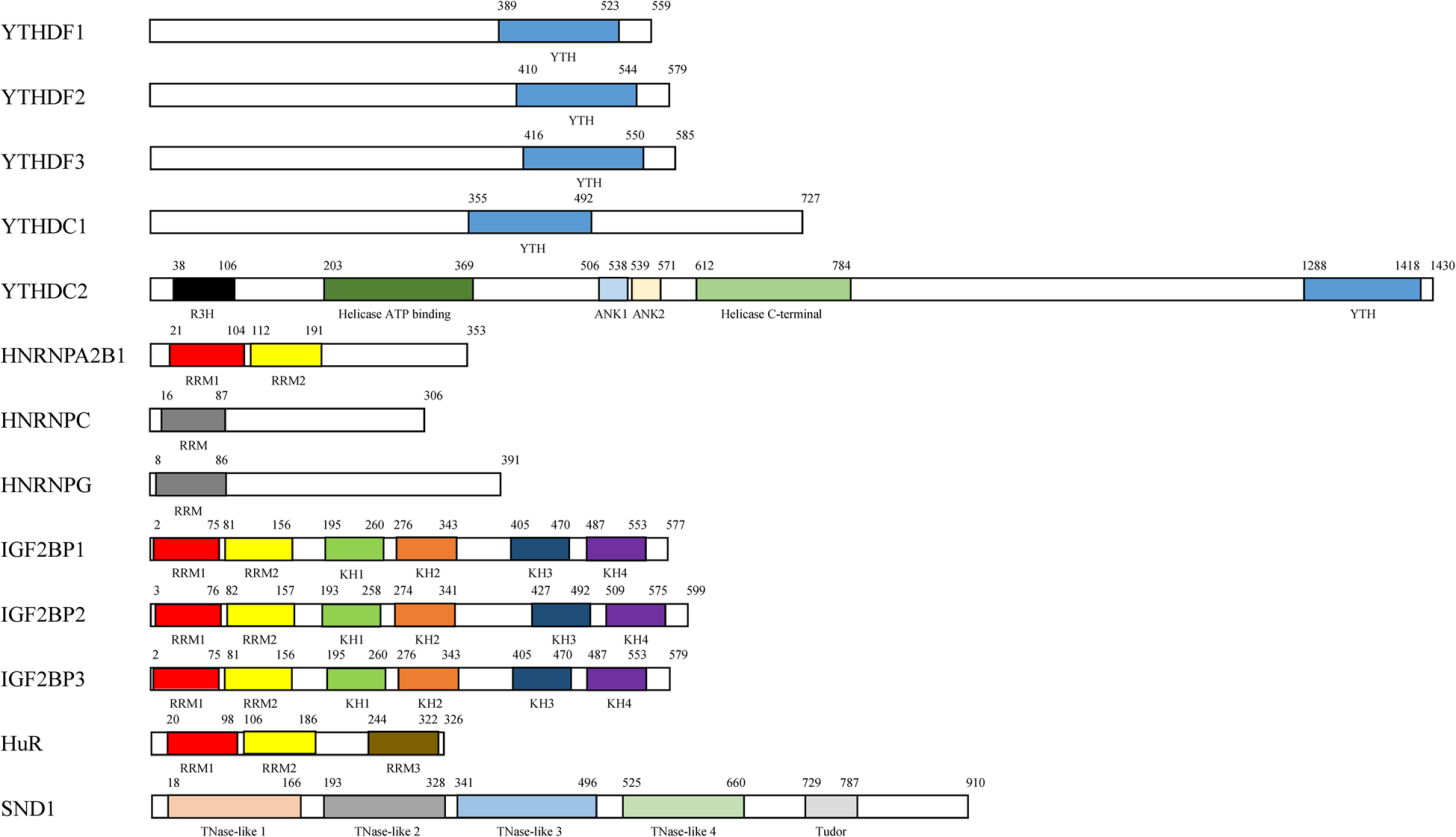
Domain architectures of m^6^A-binding proteins

**Fig. 2.**
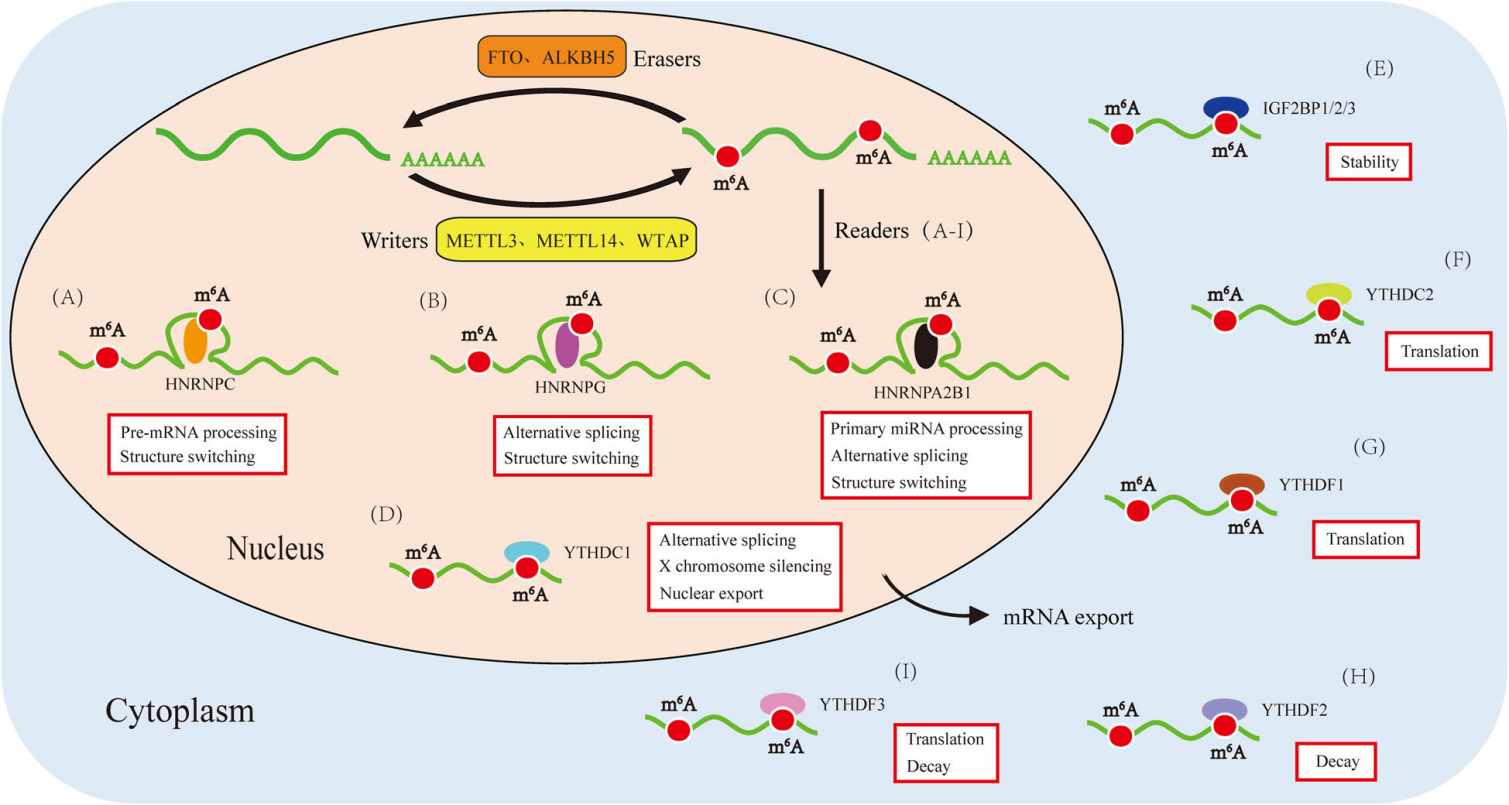
The regulation of m^6^A modification. m^6^A is established by m^6^A methyltransferases (“writers”) and removed by m^6^A demethylases (“erasers”). m^6^A readers are involved in multiple procedures of RNA metabolism through recognizing and binding to the m^6^A sites of RNAs. (**a**) HNRNPC plays an important role in the pre-mRNAs processing and structure switching. (**b**) HNRNPG modulates alternative splicing and structure switching. (**c**) HNRNPA2B1 accelerates primary miRNAs processing, alternative splicing, and structure switching. (**d**) YTHDC1 participates in the alternative splicing, nuclear export, and X chromosome silencing. (**e**) IGF2BP1/2/3 have a function to increase the stability of targeted mRNAs. (**f**) YTHDC2 promotes mRNAs translation. (**g**) YTHDF1 augments mRNAs translation. (**h**) YTHDF2 facilitates mRNAs decay. (**i**) YTHDF3 cooperates with YTHDF1 to increase mRNAs translation, and strengthens mRNAs decay mediated by YTHDF2

With the advancements in the technique of high-throughput sequencing, m^6^A modification becomes an emerging focus of investigation. Increasingly, more researches have reported the roles of different m^6^A-binding proteins. YTHDF1, YTHDF3, and YTHDC2 promote mRNA translation, while YTHDF2 and YTHDF3 facilitate mRNA decay. YTHDC1 plays an important role in the alternative splicing, nuclear export and X chromosome silencing. IGF2BP1-3 have a function to increase the stability of targeted mRNAs. In addition, HNRNP family are involved in the alternative splicing, RNA processing and structure switching. The specific structures and functions of m^6^A-binding proteins are summed up in Fig. 1 and Fig. 2, respectively. More importantly, m^6^A readers act as a promoter or inhibitor in the multiple biological processes, such as tumorigenesis (Table 2), hematopoiesis, viral replication, immune response, and adipogenesis. However, until now, the understanding about m^6^A readers is just the tip of the iceberg. There remains a lot of space for exploring in the research on m^6^A-binding proteins.

**Table 2:** The role of m^6^A-binding proteins in human solid cancers and hematological malignancy

Cancers	m^6^A readers	Target RNAs	Mechanism	Reference
Hepatocellular carcinoma	YTHDF1	Snail	Accelerating the translation of Snail mRNA	[47]
	YTHDF2	EGFR	Destabilizing EGFR mRNA	[42]
	YTHDF2	IL-11, SERPINE2	Increasing the degradation of IL-11 and SERPINE2 mRNAs	[43]
	YTHDF2	SOCS2	Facilitating SOCS2 mRNA decay	[44]
	IGF2BP1/2/3	MYC	Enhancing the expression of MYC mRNA	[32]
	IGF2BP1	SRF	Promoting SRF mRNA translation	[48]
Colorectal cancer	YTHDF1	β-catenin, WNT6, FZD9	Increasing the expression of β-catenin, WNT6 and FZD9A to activate Wnt/β-catenin pathway	[49]
	YTHDF3	GAS5	Enhancing the degradation of lncRNA GAS5	[50]
	YTHDC1	circNSUN2	Facilitating circNSUN2 export from nucleus to cytoplasm	[51]
	IGF2BP2	HMGA2	Forming a circNSUN2/IGF2BP2 complex to fortify the stability of HMGA2	[51]
	IGF2BP2	SOX2	Stabilizing SOX2 mRNA	[52]
Gastric cancer	IGF2BP1	SEC62	Augmenting SEC62 mRNA translation	[53]
	IGF2BP3	HDGF	Facilitating HDGF mRNA expression	[54]
	HuR	ZMYM1	Fortifying the stability of ZMYM1 mRNA	[116]
Lung cancer	YTHDF1	CDK2, CDK4, cyclinD1	Promoting the translations of CDK2, CDK4 and cyclinD1	[56]
	YTHDF1	Keap1	Leading to cisplatin resistance of tumor cells via modulating the Keap1-Nrf2-AKR1C1 axis	[56]
	YTHDF1/3	YAP	Up-regulating YAP expression	[57]
	YTHDF2	6PGD	Facilitating 6PGD degradation	[55]
	YTHDF3	MALAT1	Increasing MALAT1 stability	[57]
	MELLT3	EGFR, TAZ	Accelerating the translation of EGFR and TAZ	[58]
Bladder cancer	YTHDF1/3	ITGA6	Promoting ITGA6 mRNA translation	[59]
	YTHDF1	CDCP1	Enhancing the expression of CDCP1 mRNA	[60]
	MELLT3	CDCP1	Facilitating CDCP1 translation and strengthening the binding of YTHDF1 to CDCP1	[60]
Endometrial cancer	YTHDF1	PHLPP2	Increasing the expression of PHLPP2	[61]
	YTHDF2	PRR5, PRR5L, mTOR	Diminishing the abundance of PRR5, PRR5L, and mTOR	[61]
Ovarian cancer	YTHDF1	EIF3C	Targeting at EIF3C to enhance its translation efficiency	[62]
Cervical cancer	YTHDF2	GAS5	Abrogating the GAS5 expression	[63]
Melanoma	YTHDF2	PD-1 (PDCD1), CXCR4, SOX10	Downregulating the mRNA and protein levels of three key intrinsic pro-tumorigenic factors, including PD-1 (PDCD1), CXCR4 and SOX10	[64]
	YTHDF1	HINT2	Promoting the translation of HINT2 mRNA	[65]
Breast cancer	YTHDF2	BNIP3	Facilitating the degradation of BNIP3 mRNA	[66]
Pancreatic cancer	IGF2BP2	DANCR	Enhancing the DANCR expression	[67]
Acute myeloid leukemia	YTHDF2	TNFR2	Reducing the TNFR2 expression	[81]
	YTHDF2	MYC, CEBPA	Accelerating the decay of MYC and CEBPA	[82]

Firstly, a few of non-classic m^6^A readers are found through RNA sequencing, such as HuR and SND1. ZMYM1 mRNA, which could facilitate the process of EMT and the metastasis of GC, was modified by m^6^A and its stability was enhanced relying on the recognition by HuR [116]. Another novel m^6^A reader SND1 bound to the m^6^A sites of ORF50 transcript and promoted its stability. Depletion of SND1 inhibited the expression of KSHV early genes, affecting KSHV lytic replication [117]. Even so, the specific functions and mechanisms of new m^6^A-binding proteins are still not comprehensively elucidated.

Secondly, the inhibitors of m^6^A-related enzymes have been actively explored. For example, R-2HG [82] and meclofenamic acid (MA) [118] are two FTO inhibitors and have been confirmed to inhibit tumor cell growth and induce cell apoptosis. However, the inhibitors of m^6^A-binding proteins have not been identified. Actually, as the direct executers for m^6^A-dependent bioprocesses, m^6^A readers seem more indispensable for controlling multiple biological events including tumorigenesis. Therefore, further studies are needed to find the inhibitors of m^6^A readers in order to provide the novel and effective strategies for clinical therapy.

Thirdly, m^6^A exists in a broad range of viruses and is tightly associated with viral infectivity and replication. Surprisingly, the roles of m^6^A readers in the virus infections like KSHV, HIV, and IAV are sometimes contradicted with the well-recognized effects. A reasonable explanation may be that readers indirectly regulate the expression of viral genes via controlling the level of host antiviral transcripts. What is more, during the identical virus infection, even the same reader could function differentially relying on the cell context. It highlights the complexity of m^6^A-medaited impacts on virus processing which is host-dependent. Hence, it would be meaningful to systematically clarify the functions of m^6^A readers in the virus-host interaction.

Overall, m^6^A modification is emerging as a rising star in epigenetic research, and the evolving deciphering of m^6^A-binding proteins exposes a more comprehensive understanding of m^6^A methylation. The m^6^A readers are extensively responsible for multiple biological processes, and they extend the repertoire of m^6^A epitranscriptome, offering novel insights into its underlying molecular mechanisms. Apart from exploring more potential functional m^6^A-binding proteins, further investigations are required to not only exploit the complicated roles of m^6^A readers due to specific cell context, but also develop the efficient therapeutic interventions based on m^6^A readers.

## Data Availability

Not applicable.

## Abbreviations

m^6^A: N^6^-methyladenosine
YTHDF: YT521-B homology domain family
YTHDC: YT521-B homology domain containing
IGF2BP: Insulin-like growth factor 2 mRNA-binding protein
HNRNPA2B1: Heterogeneous nuclear ribonucleoproteins A2/B1
HNRNPC: Heterogeneous nuclear ribonucleoproteins C
HNRNPG: Heterogeneous nuclear ribonucleoproteins G
HCC: Hepatocellular carcinoma
CRC: Colorectal cancer
GC: Gastric cancer
HSC: Hematopoietic stem cell
AML: Acute myeloid leukemia
KSHV: Kaposi’s sarcoma-associated herpes virus
HIV: Human immunodeficiency virus
IAV: Influenza A virus
HCV: Hepatitis C virus
DC: Dendritic cell
IFN-β: Interferon-β
MCE: Mitotic clonal expansion
